# The epidemiology of hospital death following pediatric severe community acquired pneumonia

**DOI:** 10.1186/s13052-021-00966-0

**Published:** 2021-02-08

**Authors:** Xiao-Xiao Ao

**Affiliations:** Emergency department of Children’s Hospital of Chongqing Medical University, National Clinical Research Center for Child Health and Disorders, Ministry of Education Key Laboratory of Child Development and Disorders,Chongqing Key Laboratory of Pediatric, Chongqing, 400014 China

**Keywords:** Community acquired pneumonia, Pediatric, Extrapulmonary organ dysfunction, Multiple organ dysfunction syndrome

## Abstract

**Background:**

Community acquired pneumonia is the primary cause of pediatric hospitalizations and deaths in children under 5 years of age. But the epidemiology of death in pediatric severe community acquired pneumonia was not well characterized.

**Methods:**

This retrospective observational study was performed at the academic Emergency department and intensive care unit and we investigated the timing, cause, mode and attribution of death in children with severe community acquired pneumonia.

**Results:**

Of 962 subjects with severe community acquired pneumonia, there were 57 non-survivors (5.9% mortality). Median time to death was 7 [IQR 3,16] days from severe community acquired pneumonia recognition. Patients dying ≤7 days were younger, had greater illness severity and higher rate of congenital heart disease, who were more likely to die of a cardiovascular cause. Multiple organ dysfunction syndrome predominated in deaths > 7 days. Unsuccessful cardiopulmonary resuscitation was the most common mode of death at all timepoints. Our findings suggested that in pediatric severe community acquired pneumonia, early deaths were due primarily to cardiovascular dysfunction, while later deaths were more commonly due to multiple organ dysfunction syndrome.

**Conclusions:**

Deaths from non-pulmonary factors accounted for a substantial portion of non-survivors. Respiratory dysfunction accounted for only a minority of deaths. Our study highlighted limitations associated with rescuing patients with severe pneumonia from death if extrapulmonary organ dysfunctions could not be simultaneously managed.

## Introduction

Community acquired pneumonia (CAP) is the top cause of pediatric hospitalizations [1, 2]. And severe pneumonia is responsible for > 75% of these one million of the six million deaths caused by respiratory infections annually in children under 5 years of age [3]. In China, there are 21.1 million new CAP episodes in children under 5 years old annually. But epidemiology of death following childhood severe pneumonia remains poorly described.

Understanding the epidemiology of death in severe CAP is necessary to set appropriate clinical and research priorities, as children who die early may have distinct risk factors, pathophysiology, and response to therapy than those who die further out from pneumonia recognition. Later deaths may also be increasingly attributed to underlying complications rather than to pneumonia itself.

We sought to characterize the timing, cause, mode, and attribution of death in children with severe pneumonia and analyze the factors of severe pneumonia leading to death during the early or later illness course in under-five children in the current era of systematic screening and early positive therapy.

## Patients and methods

### Study location and inclusion criteria

We conducted a retrospective analysis of all patients treated for severe CAP in the emergency department (ED) and/or PICU at Children’s Hospital affiliated to Chongqing Medical University (CHCMU) who died between January 1, 2016 and December 31, 2018. Patients were included if they aged between 29 days to 5 years, met criteria for severe pneumonia as defined by the British Thoracic Society (BTS) and the Infectious Diseases Society of America (IDSA) [4, 5], were treated in either the ED or PICU, invasively ventilated, and died prior to hospital discharge. The Institutional Review Boards at CHCMU approved this study under a waiver of informed consent.

### Data collection and statistical analysis

A detailed review of the medical record was completed for all severe CAP non-survivors by two investigators independently. Data were collected about demographics, comorbid conditions, microbiology, organ dysfunction, cause and mode of death. Cause of death was assigned to one of three exclusive categories: 1) acute respiratory distress syndrome (ARDS), 2) cardiovascular dysfunction, or 3) multiple organ dysfunction syndrome (MODS). Mode of death was assigned one of two exclusive categories: 1) withdrawal or withholding of life-sustaining therapies, or 2) unsuccessful cardiopulmonary resuscitation (CPR). ARDS met the definition of the Pediatric Acute Lung Injury Consensus Conference (PALICC) [6]. Organ dysfunction was defined using the consensus criteria for pediatric sepsis and MODS was defined as ≥2 concurrent organ system dysfunctions [7]. Severity of illness was determined by the Pediatric Risk of Mortality (PRISM) III at 12 h after admission. Timing of death was calculated as the number of calendar days from admission and stratified into ≤7 days (early deaths) and > 7 days (later deaths) after severe CAP recognition. Severe pneumonia recognition to death with one day assigned to those who died on the day of hospital presentation. For patients who met criteria for severe pneumonia on arrival, day of severe pneumonia recognition was defined as the day of hospital presentation.

Analyses were performed using SPSS Version 24.0. Descriptive data were presented as means ± standard deviation (SD) for normally-distributed variables, medians with interquartile ranges (IQR) for non-parametric continuous variables, and frequencies with percentages for categorical variables. Mann-Whitney non-parametric tests and chi-square tests were used to compare continuous and categorical variables, respectively. Statistical significance was defined at *p* < 0.05.

## Results

**Fig. 1:**
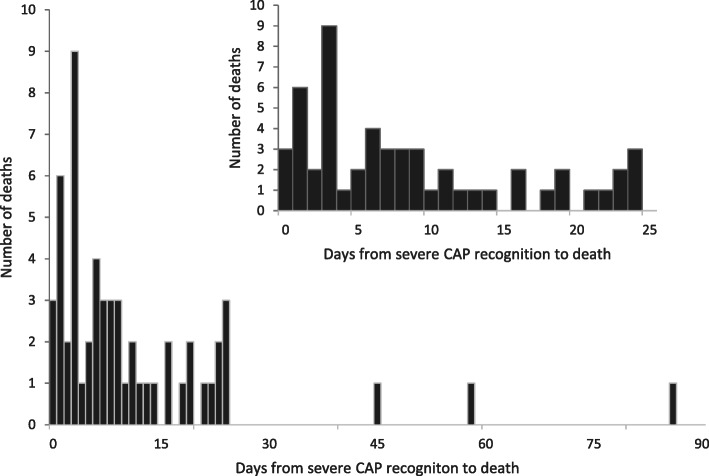
Days from severe CAP recognition to death. Frequency histogram of days from severe CAP recognition to death. Inset shows timing of death within first 25 days following severe CAP recognition with each bin equal to one day

During the two-year study period, of 962 subjects with severe CAP, a total of 57 children aged between 29 days old to 5 years old were included in our analysis, who were treated for severe CAP in the ED and/or PICU and died prior to hospital discharge. Patient characteristics by time from severe CAP recognition to death were shown in Table 1. The time to death from severe CAP recognition was a median of 7 [IQR 3,16] days (Fig. 1). Patients who died ≤7 days from severe CAP recognition were younger, had higher PRISM III scores and more congenital heart disease (CHD) than patients who died at later times.

**Table 1:** Demographics of the non-survivors

		Days between Severe CAP Recognition and Death		
Variable	All	≤ 7d	> 7d	***P***-value
n	57	20	37	
Age, months (median [IQR])	6 [4,9]	3.5 [1.8,5]	8 [6,13]	< 0.001
Female (%)	28 (49.1)	11 (55.0)	17 (45.9)	0.514
PRISM III (median [IQR]) ^1^	16 [13.3,21]	23 [21,26]	15 [12,16]	< 0.001
Comorbidity (%)				
CHG	20 (35.1)	12 (60.0)	8 (21.6)	0.004
Respiratory dysplasia	7 (12.3)	3 (15.0)	4 (10.8)	0.970
Moderate/severe malnutrition ^2^	11 (19.3)	6 (30.0)	5 (13.5)	0.249
Known chromosomal abnormality	1 (1.8)	1 (5.0)	0	
Neurologic disorder	1 (1.8)	1 (5.0)	0	
Immunocompromised	1 (1.8)	1 (5.0)	0	
Prematurity	6 (10.5)	3 (15.0)	3 (8.1)	0.721
Cause of death (%)				0.035
ARDS	7 (12.3)	3 (15.0)	4 (10.8)	
Cardiovascular dysfunction	23 (40.4)	12 (60.0)	11 (29.7)	
MODS	27 (47.4)	5 (25.0)	22 (59.5)	
Mode of death (%)				0.167
Withdrawal/withholding therapies	18 (31.6)	4 (20.0)	14 (37.8)	
Unsuccessful CPR	39 (68.4)	16 (80.0)	23 (62.2)	
Hematologic	9 (15.8)	2 (10.0)	7 (18.9)	0.617
Pathogen (%)				
Bacterial	24 (42.1)	9 (45.0)	15 (40.5)	0.745
Viral	17 (29.8)	5 (25.0)	12 (32.4)	0.558
Fungal	3 (5.3)	0	3 (8.1)	0.492
Unknown	26 (45.6)	10 (50.0)	16 (43.2)	0.625
**Days between Severe CAP Recognition and Death**				
**Variable**	**All**	**≤ 3d**	**> 3d**	**P-value**
Complicated infection	13 (22.8)	4 (20.0)	9 (24.3)	0.968

IQR, interquartile range; PRISM, pediatric risk of mortality; CHG, congenital heart disease; ARDS, acute respiratory distress syndrome; MODS, multiple organ dysfunction syndrome; CPR, cardiopulmonary resuscitation

1 Data available for 54 patients due to lack of scoring for deaths prior to 12 h after ED/PICU admission

2 Moderate/severe malnutrition was defined as weight-for-age less than -2SD from means

The most common cause of death was MODS (47.4%) in all non-survivors, followed by cardiovascular dysfunction (40.4%), and the cause of death varied significantly at different time points following severe CAP recognition. MODS predominated as cause of death > 7 days (59.5%) from severe CAP recognition while accounted for 25.0% deaths within seven days. 60.0% of early deaths ≤7 days from severe CAP recognition due to cardiovascular dysfunction compared to 29.7% in patients who died after seven days. (*p* < 0.05, Table 1).

Unsuccessful CPR was the most common mode of death, accounting for 68.4% of all deaths. 31.6% died following withdrawal or withholding of therapies. Mode of death didn’t vary significantly at different time points following severe CAP recognition (Table 1). 80.0% of deaths ≤7 days and 62.2% of deaths > 7 days from severe CAP recognition occurred after unsuccessful CPR, respectively.

Organ dysfunction at time of death was not uncommon in all non-survives. Table 2 showed the proportion with organ dysfunction on the day of death. Cardiovascular dysfunction was more common in patients dying ≤7 days from severe CAP recognition. Respiratory dysfunction including ARDS was more common in patients dying > 7 days (*p* < 0.05).

**Table 2:** Organ dysfunctions at death

		Days between Severe CAP Recognition and Death		
	All	≤ 7d	> 7d	P-value
ARDS	30 (52.6)	5 (25.0)	25 (67.6)	0.002
Cardiovascular	31 (54.4)	16 (80.0)	15 (40.5)	0.004
Neurologic	6 (10.5)	1 (5.0)	5 (13.5)	0.584
Renal	5 (8.8)	1 (5.0)	4 (10.8)	0.803
Hepatic	7 (12.3)	2 (10.0)	5 (13.5)	1.000
Hematologic	9 (15.8)	2 (10.0)	7 (18.9)	0.617

ARDS, acute respiratory distress syndrome

## Discussion

It was estimated that 7–13% of the 156 million of pneumonia cases occurred annually around the world might progress to severe cases, and there had been many studies on the risk predictors of pneumonia severity. But the risk factors associated with timing of death and the epidemiology of cause of death of severe CAP were less explored.

According to the previous investigation, the risk factors to increase the severity of pneumonia were different between developed countries and countries with less resource. In deprived areas, female sex, age < 2 months, prematurity, severe malnutrition and chronic comorbidity were known risk factors [8–10]. While in developed countries, the incidence of these risk factors was much smaller, and the presence of altered mental status, signs and symptoms of pneumonia were identified as risk factors for severe CAP [11]. In this study, we also considered above risk factors for disease severity to determine whether they had impacts on death. Our finding that patients dying ≤7 days from severe CAP recognition were younger, had CHD or more comorbidities, and predominately died from cardiovascular dysfunction was consistent with prior studies. Although infants with pneumonia were prone to airway obstruction due to the airway anatomical and physiological characteristics, it was easy to improve ventilation through current clinical measures and be less likely to die of airway obstruction. Risk factors for severe pneumonia, such as CHD, due to abnormal cardiovascular structure and function, abnormal blood flow distribution, hypoxic acidosis, etc. resulted in heart failure, shock and other poor prognosis [12].

Zhang et al. found that older children with lung infection might be less likely to have airway obstruction as their respiratory systems developed and more likely to appeared hypoxemia and ARDS. In our study, later deaths > 7 days from severe CAP recognition with older age predominately due to MODS exhibited a higher rate of ARDS. Similar findings were noted in previous literatures. In patients with lung injury might developed MODS and ARDS was an independent risk factor for MODS [13]. Primary hypoxemia and pulmonary biotrauma might contribute to extrapulmonary organ dysfunction [14].

Moreover, deaths from cardiovascular cause were comparable to that from MODS. Mortality in severe CAP appeared to be primarily related to non-pulmonary organ failure. The predominance of cardiovascular and MODS as causes of deaths in our study reinforce that the treatment for severe pneumonia should be considered in the context of a broader systemic illness and improving lung disease could prove inadequate to rescue patients from death if extrapulmonary organ dysfunctions could not be simultaneously managed.

In prior studies of pediatric ARDS, sepsis and all PICU deaths, withdrawal or withholding of therapies was the common mode of death [15–17], and patients who died at later times were unlikely to be previously healthy. But patients dying at later times in our observation were more likely to be previously healthy. This might help explain why the mode of death in our study differed from those in other studies. While we were not able to discern the underlying reasons in our study, patients for this mode of death of unsuccessful CPR suggested that previously healthy contributed to these decisions not to give up treatments.

Our study had limitations. Because our data were limited to in-hospital deaths, we could not determine if our findings were representative of the epidemiology of post-discharge mortality. In addition, our study reflected the practices of an academic children’s hospital, it was not clear how generalizable our findings were to other settings. Finally, the retrospective and subjective nature of assigning cause of death and organ dysfunctions at death could introduce misclassification bias.

## Conclusion

In severe CAP, early deaths were due primarily to cardiovascular dysfunction with younger age and a higher rate of CHG as comorbidities, while later deaths were more likely due to MODS. Organ dysfunctions was common at time of death. Future research priorities in pediatric severe CAP should include determining risk factors for early deaths and consider that life-saving interventions may need to be differentially targeted based on timing and cause of death.

## Data Availability

The datasets used and/or analyzed during the current study are available from the corresponding author on request.

## Abbreviations

CAP: community acquired pneumonia
ED: emergency department
ARDS: acute respiratory distress syndrome
MODS: multiple organ dysfunction syndrome
CPR: cardiopulmonary resuscitation
PRISM: the Pediatric Risk of Mortality
CHD: congenital heart disease
